# The first complete mitochondrial genome data of *Hippocampus kuda* originating from Malaysia

**DOI:** 10.1016/j.dib.2020.105721

**Published:** 2020-05-21

**Authors:** Puteri Nur Syahzanani Jahari, Nur Fatihah Abdul Malik, Mohd Shahir Shamsir, M. Thomas P. Gilbert, Faezah Mohd Salleh

**Affiliations:** a Department of Biosciences, Faculty of Science, Universiti Teknologi Malaysia, 81310 Johor Bahru, Johor, Malaysia; b Johor Biotechnology & Biodiversity Corporation (J-Biotech), Level 2, Bio-XCell Malaysia, No. 2, Jalan Bioteknologi 1, SiLC Industrial Park, 79200 Iskandar Puteri, Johor, Malaysia; c Faculty of Applied Sciences and Technology, Universiti Tun Hussein Onn Malaysia, Pagoh Higher Education Hub, 84600 Muar, Johor, Malaysia; d Section for Evolutionary Genomics, The GLOBE Institute, University of Copenhagen, Øster Farimagsgade 5a, 1353, Copenhagen, Denmark

**Keywords:** *Hippocampus kuda*, mitogenome, *H. kuda* clade

## Abstract

The spotted seahorse, *Hippocampus kuda* population is exponentially decreasing globally due to habitat loss contributed by massive coastal urbanization as well as its large exploitation for Chinese herbal medicine. Genomic data would be highly useful to improve biomonitoring of seahorse populations in Malaysia via the usage of non-invasive approaches such as water environmental DNA. Here we report the first complete mitogenome of two *H. kuda* individuals originating from Malaysia, generated using BGISEQ-500RS sequencer. The lengths of both mitogenomes are 16,529bp, consisting of 13 protein-coding genes, 22 transfer RNA genes, two ribosomal RNA genes, and a control region. The overall base composition was 32.46% for A, 29.40% for T, 14.73% for G and 23.41% for C with AT rich features (61.86%). The gene organization of Malaysian *H. kuda* were similar to that of most teleost species. A phylogenetic analysis of the genome against mtDNA data from other *Hippocampus* species showed that Malaysian *H. kuda* samples clustered with *H. capensis, H. reidi* and *H. kuda*. Notably however, analysis of the data using BLASTn revealed they had 99.18% similarity to *H. capensis*, and only 97.66% to *H. kuda* and *H. reidi*, which are all part of the unresolved *H. kuda* complex. The mitogenomes are deposited in Genbank under the accession number MT221436 (HK1) and MT221436 (HK2).

Specifications Table

**Table:** 

Subject	Genomics
Specific subject area	Mitogenomics
Type of data	Mitogenome sequence data in FASTA file format, tables, mitogenome map in figure format (.PNG), phylogenetic tree in figure format (.PNG) and newick format (.nwk)
How data were acquired	BGISEQ-500RS High-throughput sequencing kit (PN: 85–05238-01, BGI)
Data format	Raw and analyzed
Parameters for data collection	A small amount of tissue from the tip of the tail of Hippocampus kuda complex was sampled, genomic DNA was extracted using Qiagen Blood and Tissue Kit (Qiagen, Valencia, CA), hardware used for quality check includes Qubit 2.0 Fluorometer and Agilent 2100 Bioanalyzer, library preparation prior to sequencing required DNA fragmentation using M220 Focused-ultrasonicator (Covaris, USA), the sample was sequenced using BGISEQ-500RS High-throughput sequencing kit (PN: 85–05238-01, BGI).
Description of data collection	The complete mitogenomes were assembled by using MITOBIM v1.8. The mitogenome mapping quality was assessed using PALEOMIX. The mitogenomes were annotated using MitoAnnotator and GB2sequin annotation web application. The circular mitochondrial genome map was drawn using OGDRAW. Phylogenetic relationship between *Hippocampus* sp. was constructed using MEGAX.
Data source location	These individuals were caught as incidental catch in fisherman nets at Pulai River, Johor, Malaysia (Latitude: 1° 22′ 59.99" N Longitude: 103° 31′ 59.99" E)
Data accessibility	The mitogenome data is available in Genbank with the accession numbers MT221436.1 (https://www.ncbi.nlm.nih.gov/nuccore/MT221436.1) and MT221437.1 (https://www.ncbi.nlm.nih.gov/nuccore/MT221437.1) and Mendeley data (http://dx.doi.org/10.17632/b3yjvcn7k2.1) [1].
Related research article	S.A. Lourie, R.A. Pollom, S.J. Foster, A global revision of the Seahorses Hippocampus Rafinesque 1810 (Actinopterygii: Syngnathiformes): Taxonomy and biogeography with recommendations for further research, Zootaxa. 4146 (2016) 1–66.

## Value of the Data

• The mitogenomes will be useful for *H. kuda* species monitoring using water environmental DNA approach
• The data generated will be useful to resolve the *H. kuda* complex phylogenetic, population and evolutionary studies.
• The data will contribute to our understanding of any adaptive introgression which take place within the *H. kuda* clade.

## 1 Data Description

The spotted seahorse, *Hippocampus kuda* Bleeker, 1852a is known for its species-complex due to the exceptionally large distribution all around the world [2]. However, among the *Hippocampus* genus, this species is decreasing due to overexploitation for its alleged medicinal properties [3]. Globally, seahorse populations are suffering an exponential decline due to anthropogenic and environmental actions that threaten their survival [3]. Massive development of coastal areas in Malaysia for mega urbanization projects, in regions that serve as its natural habitat are also clearly a threat for its populations [4]. Currently, this species is listed as vulnerable under the (IUCN) Red List of Threatened Species [5].

**Fig. 1:**
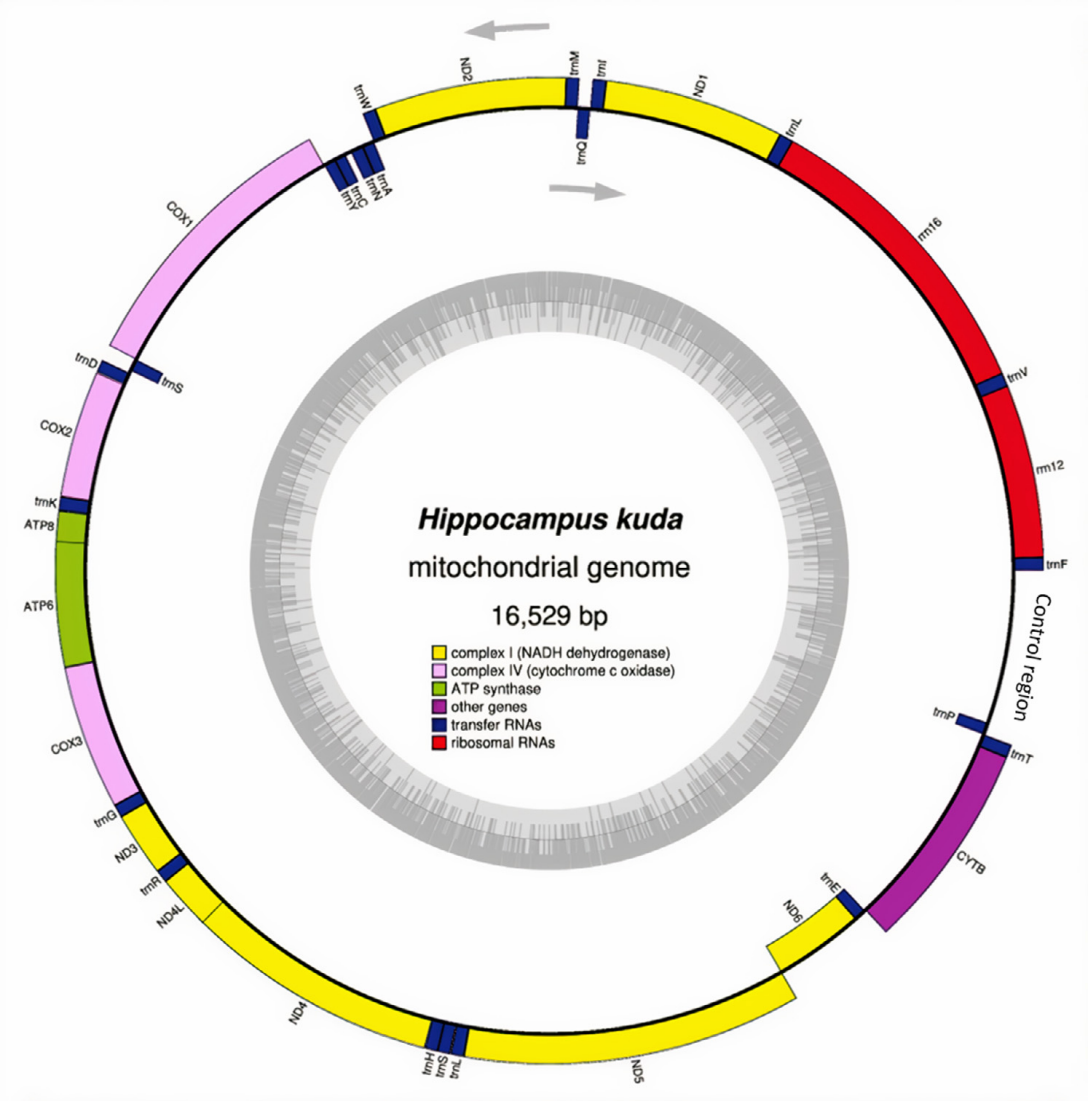
Map of the *Hippocampus kuda* mitochondrial genome. Genes encoded by the heavy strand shown outside the circle, and those encoded by the light strand are shown inside. The direction of the arrows shows the genes inside the circle are transcribed clockwise and genes outside the circle transcribed counter clockwise. The inner ring shadow indicates the GC content of the genome.

Here, we provide the Malaysian *H. kuda* mitogenomes with 16,529bp in length. The data information for each individual is presented in Table 1. The representative complete mitogenome map in Fig. 1 shows similar gene arrangement containing 37 genes; 13 protein-coding genes (PCGs), 22 tRNA genes, two rRNA genes, and a non-coding A+T rich control region (D-loop) as in other seahorse mitogenomes [6]. Total length of 13 PCGs is 11,319 bp and they encoded 3773 amino acids. The overall base composition is estimated to be 32.46% for A, 29.40% for T, 14.73% for G and 23.41% for C, indicating an obvious AT rich feature (61.86%). The genes of NAD6 and eight tRNAs are encoded on the light strand, while the rest of mitochondrial genes are encoded on H-strand (Table 2, supplementary data 1).

**Table 1:** Sequencing data for each *H. kuda* individual

	*Hippocampus* kuda (HK1)	*Hippocampus* kuda (HK2)
Trimmed reads	119,174,870	34,830,940
Mapped reads	17293	25180
% Mapped reads	0.01	0.07
Depth of coverage (x)	63.19	84.43

**Table 2:** Features of the mitochondrial genome of *Hippocampus kuda*

Gene	Position		Size (bp)	Amino acid	Strand
	From	To			
*tRNA(Phe)*	1	71	71		H
*12S rRNA*	72	1009	938		H
*tRNA (Val)*	1010	1082	73		H
*16S rRNA*	1083	2778	1696		H
*tRNA (Leu)*	2779	2852	74		H
*NAD1*	2853	3827	975	325	H
*tRNA (Ile)*	3829	3900	72		H
*tRNA (Gln)*	3900	3970	71		L
*tRNA (Met)*	3972	4041	70		H
*NAD2*	4042	5080	1039	346	H
*tRNA (Trp)*	5081	5151	71		H
*tRNA (Ala)*	5153	5221	69		L
*tRNA (Asn)*	5223	5295	73		L
*tRNA (Cys)*	5331	5396	66		L
*tRNA (Tyr)*	5397	5463	67		L
*COXI*	5465	7018	1554	518	H
*tRNA (Ser)*	7020	7090	71		L
*tRNA (Asp)*	7105	7172	68		H
*COX2*	7177	7867	691	230	H
*tRNA (Lys)*	7868	7942	75		H
*ATP8*	7944	8111	168	56	H
*ATP6*	8102	8784	683	228	H
*COX3*	8785	9568	784	261	H
*tRNA (Gly)*	9569	9638	70		H
*NAD3*	9639	9987	349	116	H
*tRNA (Arg)*	9988	10056	69		H
*NAD4-L*	10057	10353	297	99	H
*NAD4*	10347	11725	1379	460	H
*tRNA (His)*	11728	11796	69		H
*tRNA (Ser)*	11797	11864	68		H
*tRNA (Leu)*	11867	11939	73		H
*NAD5*	11940	13775	1836	612	H
*NAD6*	13772	14293	522	174	L
*tRNA (Glu)*	14294	14362	69		L
*COB*	14367	15507	1141	380	H
*tRNA (Thr)*	15508	15579	72		H
*tRNA (Pro)*	15579	15648	70		L
Control region	15648	16529	882		

**Fig. 2:**
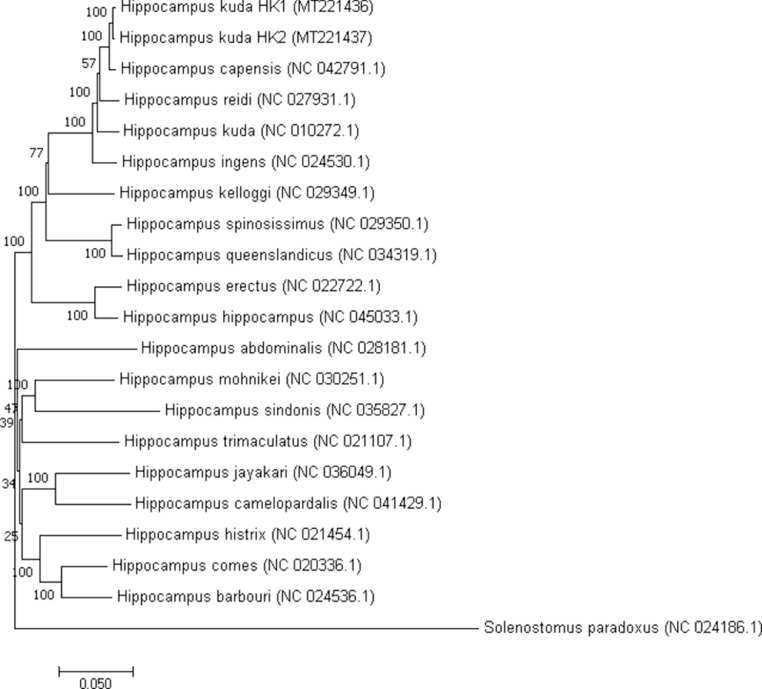
Phylogenetic tree of two Malaysian *H. kuda* (MT221436 and MT221436) and 18 *Hippocampus* genus constructed with the combined protein-coding gene nucleotide sequences using MEGAX [24]. The tree was generated from NJ method using pipefish as an outgroup. Bootstrap values generated from 1000 replicates for NJ analysis. The number at each node indicated the bootstrap probability of NJ analysis.

A phylogenetic tree of all available *Hippocampus* mitogenomes was also constructed (Fig. 2). In total we included the eighteen *Hippocampus* species available in Genbank along with both Malaysian *H. kuda* generated in this work. The mitogenomes were firstly aligned using MUSCLE [7], after which a phylogenetic tree was constructed using the neighbor-joining (NJ) method. The 18 mitogenomes include; *Hippocampus kuda* (accession no. NC_010272.1), *Hippocampus comes* (accession no. NC_020336.1), *Hippocampus trimaculatus* (accession no. NC_021107.1), *Hippocampus histrix* (accession no. NC_021454.1), *Hippocampus erectus* (accession no. NC_022722.1), *Hippocampus ingens* (accession no. NC_024530.1), *Hippocampus barbouri* (accession no. NC_024536.1), *Hippocampus reidi* (accession no. NC_027931.1), *Hippocampus abdominalis* (accession no. NC_028181.1), *Hippocampus kelloggi* (accession no. NC_029349.1), *Hippocampus spinosissimus* (accession no. NC_029350.1), *Hippocampus mohnikei* (accession no. NC_030251.1), *Hippocampus queenslandicus* (accession no. NC_034319.1), *Hippocampus sindonis* (accession no. NC_035827.1), *Hippocampus jayakari* (accession no. NC_036049.1), *Hippocampus camelopardalis* (accession no. NC_041429.1), *Hippocampus capensis* (accession no. NC_042791.1), *Hippocampus hippocampus* (accession no. NC_045033.1). A mitogenome of a pipefish, *Solenostomus paradoxus* (accession no. NC_024186.1) was selected as an outgroup. The phylogenetic tree indicates that the Malaysian *H. kuda* (HK1 and HK2) firstly clustered with *H. capensis*, followed by *H. reidi* and *H. kuda*.

We also compared the mitogenomes to Genbank using BLASTn, and found the closest match for both Malaysian *H. kuda* mitogenomes was a 99.18% similarity to a *H. capensis* (NC_042791.1) sample collected from Bozhou Chinese herbal medicine market (Bozhou, China) [8]. The next closest match, at 97.66%, was to the sole *H. kuda* mitogenome currently available in Genbank (NC_010272.1), from a sample originating Vancouver Aquarium, Canada [9]. A similar match was also found to a *H. reidi* sample (NC_027931.1) [10]. Interestingly, both *H. capensis* and *H. reidi* species are not found in the Malaysian region. However, it is worth noting that there is an ongoing debate about these species being associated with the unresolved ‘H. *kuda* clade’. Due to their large distribution, these species exhibit localized haplotypes, phylogeographic structuring, and variable morphology [2]. These findings clearly underscore for future studies using nuclear DNA (nuDNA) to fully resolve the relationship within these *Hippocampus* species.

## 2 Experimental Design, Materials, and Methods

### 2.1 Biological samples

Two individuals of *Hippocampus kuda* were caught incidentally as bycatch at Pulai River, Johor, Malaysia (Latitude: 1° 22′ 59.99" N Longitude: 103° 31′ 59.99" E), and identified based on its morphometric features [2]. Tissue samples of the two individuals, *H. kuda* (HK1) and *H. kuda* (HK2) were collected from the tip of the tails. The genomic DNA was extracted using Qiagen Blood and Tissue Kit (Qiagen, Valencia, CA). The DNA was later fragmented into 300-400bp using a M220 Focused-ultrasonicator (Covaris, USA) [11] and BGISeq compatible shotgun sequencing libraries were build using the Blunt-End-Single-Tube (BEST) library protocol [12]. Quantitative PCR was performed prior to index PCR in order to ensure the library was not over-amplified prior to sequencing. Each library was purified using Solid-Phase Reversible Immobilization (SPRI) bead solution. The quality control of generated libraries was quantified quantitatively and qualitatively using Qubit 2.0 Fluorometer (Invitrogen, Merelbeke, Belgium) and Agilent 2100 Bioanalyzer (Agilent, Santa Clara, USA). The libraries were pooled to the equimolar with 15 other libraries (not related to this work). Next, the libraries were sent for shotgun sequencing on the BGISEQ-500 platform in 100bp paired-end mode (PE100) (BGI, Shenzhen, China). The data generated were firstly demultiplexed by index prior to mitogenome construction.

### 2.2 Complete mitogenome generation

The quality of the raw reads generated was verified using the fastQC program (https://www.bioinformatics.babraham.ac.uk/projects/fastqc/). The raw reads were trimmed for sequencing adapters, low-quality stretches, and leading/tailing Ns using AdapterRemoval v2.2.2 [13]. Forward and reverse reads were interleaved into a single file prior to the assembly. The assembly *H. kuda* (HK1) and *H. kuda* (HK2) was conducted using MITOBIM v1.8 [14] (default k-mer size of 31), which performs reference assemblies using MIRA iterations [15]. The reference sequence used for the assembly was *H. kuda* from Vancouver Aquarium, Canada (Genbank Accession Number: NC_010272.1). Next, we used the PALEOMIX v1.2.6 BAM pipeline [16] with default parameters to remove reads shorter than 25 bp after trimming. The trimmed reads were aligned using Burrows-Wheeler Aligner [17] against the newly assembled mitogenome constructed by MITOBIM. Further trimming for the alignments that showed PCR duplicates and low-quality scores were conducted using MarkDuplicates program from Picard tools [18]. Next, the IndelRealigner tool from the Genome Analysis Toolkit (GATK) [19] was used to locally realign the reads around the small insertions and deletions (indels) in order to improve overall genome quality. Post-analysis, the statistics of the sequencing data for each individual was generated as displayed in Table 1. Tablet software [20] was used to manually check the indels and read coverage along the assembled mitogenomes. The mitogenome was annotated using the MitoAnnotator [21] and GB2sequin annotation web application [22]. The circular mitochondrial genome map was drawn using OGDRAW [23] (Fig. 1).

## Declaration of Competing Interest

The authors declare that they have no known competing financial interests or personal relationships which have, or could be perceived to have, influenced the work reported in this article.

## Appendix A. Supplementary data 1

Features of the mitochondrial genome of *Hippocampus kuda*

## Appendix B Supplementary materials

Image, application 1

Image, application 2
